# Safety and Outcomes of Catheter Ablation for Consecutive Atrial Tachycardia in Elderly Patients After Previous Cardiac Interventions

**DOI:** 10.3390/jcm14030675

**Published:** 2025-01-21

**Authors:** Ann-Kathrin Kahle, Fares-Alexander Alken, Katharina Scherschel, Ernan Zhu, Melanie A. Gunawardene, Andreas Metzner, Stephan Willems, Christian Meyer

**Affiliations:** 1 Division of Cardiology, Angiology, Intensive Care Medicine, EVK Düsseldorf, cNEP, cardiac Neuro- and Electrophysiology Research Consortium, Kirchfeldstrasse 40, 40217 Düsseldorf, Germany; ann-kathrin-kahle@gmx.de (A.-K.K.);; 2 Department of Cardiology, Pulmonology and Vascular Medicine, Medical Faculty, University Hospital Düsseldorf, Moorenstrasse 5, 40225 Düsseldorf, Germany; 3 Institute of Neural and Sensory Physiology, cNEP, cardiac Neuro- and Electrophysiology Research Consortium, Medical Faculty, Heinrich Heine University Düsseldorf, Universitätsstrasse 1, 40225 Düsseldorf, Germany; 4 Cardiovascular Research Institute Düsseldorf (CARID), Medical Faculty and University Hospital Düsseldorf, Heinrich Heine University, Moorenstrasse 5, 40225 Düsseldorf, Germany; 5 Department of Cardiology and Internal Intensive Care Medicine, Asklepios Hospital St. Georg, Lohmühlenstrasse 5, 20099 Hamburg, Germany; 6 Faculty of Medicine, Semmelweis University, 1085 Budapest, Hungary; 7 German Center for Cardiovascular Research (DZHK), Partner Site Hamburg/Kiel/Lübeck, 20251 Hamburg, Germany; 8 Department of Cardiology, University Heart and Vascular Center Hamburg, University Hospital Hamburg-Eppendorf, Martinistrasse 52, 20246 Hamburg, Germany

**Keywords:** atrial fibrillation, atrial tachycardia, catheter ablation, elderly patients, electroanatomical mapping

## Abstract

**Background:** Age is a relevant risk factor for the development of atrial arrhythmias and an independent predictor of adverse cardiovascular outcomes. The incidence of atrial tachycardia (AT) is known to increase with aging, but so far, there are no data on elderly patients with AT. Therefore, we sought to assess the safety and outcomes of AT ablation in patients ≥75 years compared to those <75 years. **Methods:** A total of 420 consecutive patients undergoing AT ablation after previous cardiac interventions (mean 2.1 ± 0.1 prior ablation procedures) were analyzed. Safety, as well as acute and mid-term outcomes of AT ablation were compared between 140 patients ≥75 years (mean age 78.1 ± 0.2 years, 22.9% aged ≥80 years (range 80–86 years)) and 280 patients <75 years (mean age 62.2 ± 0.6 years). **Results:** Patients ≥75 years were more often female (54.3% vs. 38.2%; *p* = 0.0024) and presented with more cardiac comorbidities, including arterial hypertension (85.0% vs. 64.3%; *p* < 0.0001) and coronary artery disease (33.6% vs. 18.2%; *p* = 0.0006). Acute success of AT ablation was reached in 96.4% vs. 97.9% of patients (*p* = 0.5173). Major complications (1.4% vs. 0.7%; *p* = 0.6035) and duration of hospital stay (2 (IQR 2–4) days vs. 2 (IQR 2–3) days; *p* = 0.9125) did not differ significantly between groups. During a follow-up of 364 (IQR 183–729.5) days, arrhythmia recurrences occurred in 45.0% vs. 49.3% (*p* = 0.4684), whereas repeat ablation was less frequently performed in patients ≥75 years (25.7% vs. 36.1%; *p* = 0.0361). **Conclusions:** AT ablation in patients ≥75 years after previous cardiac interventions in tertiary arrhythmia centers is safe and effective. Therefore, AT ablation should not be ruled out in elderly patients due to age alone, but should be considered based on arrhythmia burden, symptom severity and concomitant clinical and procedural risk factors.

## 1. Introduction

Age is a relevant risk factor for the development of atrial arrhythmias and an independent predictor of adverse cardiovascular outcomes [1,2,3]. The incidence of atrial tachycardia (AT) is known to increase with aging due to altered atrial remodeling [4], with typical atrial flutter ranging from 5/100,000 in patients aged <50 years to 587/100,000 in those aged ≥80 years [5,6]. Additionally, based on recent observations supporting early rhythm control of atrial fibrillation (AF) [7,8,9], the number of AF ablation procedures is expected to rise during the next decades. Consequently, consecutive AT occurring after previous cardiac interventions are of growing importance in clinical practice considering the limitations of antiarrhythmic drugs [9,10], especially in the aging population. Previous reports have demonstrated that catheter ablation for AF is safe and efficient in elderly patients but may be associated with a higher rate of arrhythmia recurrences, as well as procedural complications [11,12,13,14,15]. Despite the emerging clinical relevance, there are no data on elderly patients with AT so far [16]. Therefore, the present study aimed to assess safety and outcomes of catheter ablation for consecutive AT in patients ≥75 years after previous cardiac interventions compared to those <75 years.

## 2. Materials and Methods

### 2.1. Study Design

In this observational study, 420 consecutive patients undergoing catheter ablation for consecutive AT occurring after previous cardiac interventions, including catheter ablations and/or cardiac surgeries, were analyzed. Safety, as well as acute and mid-term outcomes of AT ablation were compared between 140 patients ≥75 years and 280 patients <75 years.

### 2.2. Electrophysiological Study

Transesophageal echocardiographic imaging was performed for exclusion of an intracardiac thrombus. Oral anticoagulation and antiarrhythmic drugs were not interrupted prior to the procedure. Femoral venous puncture was as a standard guided by manual palpation. Mapping and catheter ablation were conducted under general anesthesia or deep sedation, as previously described [17]. A steerable 6-F decapolar diagnostic catheter (Inquiry^TM^, 2–5–2 mm spacing, St. Jude Medical, St. Paul, MN, USA; or Dynamic DECA, Bard Electrophysiology, Boston Scientific, Marlborough, MA, USA) was placed in the coronary sinus as a reference, and an open-irrigated 3.5 mm tip catheter (Thermocool^®^, D- or F-Type-Curve; Biosense Webster, Irvine, CA, USA; or IntellaNav MiFi OI^®^; Boston Scientific; or IntellaNav Stablepoint^TM^; Boston Scientific) was used for ablation. Electroanatomical mapping was performed with a multielectrode catheter (Orion^TM^; Boston Scientific or Pentaray; Biosense Webster or Lasso catheter; Biosense Webster). The left atrium was accessed with fluoroscopic guidance by double transseptal access after a single transseptal puncture using a fixed-curve long sheath (SL0 or SL1, 8-F; St. Jude Medical). Heparin was applied intravenously to maintain an activated clotting time > 300 s, with the first dose administered after transseptal puncture.

For patients in sinus rhythm at the beginning of the procedure, programmed stimulation or burst atrial pacing was performed [18]. The clinical AT was assumed when cycle length and P wave morphology matched the preprocedural 12-lead electrocardiogram documentation.

### 2.3. Electroanatomical Mapping

Activation and voltage mapping was performed for all inducible AT [18]. Maps were considered complete when the entire chamber anatomy was reconstructed with the best achievable electrode–tissue contact. Activation maps were created under standard automatic beat acceptance criteria. Wavefront propagation, activation patterns, areas of slow conduction, anatomical and functional barriers, and lines of block were reviewed to target the critical isthmus or site of earliest activation [19]. Additional entrainment mapping was performed at the operator’s discretion.

### 2.4. Catheter Ablation

Radiofrequency current was applied using a maximum power of 40 W and peak tip temperature of 42 °C. For macro-reentrant tachycardia, the critical isthmus was targeted in order to transect the circuit by linear ablation. Localized reentrant AT were targeted at their critical site, and focal AT by ablation at the site of earliest activation. Endpoints of previously performed ablations, such as pulmonary vein isolation, or the integrity of lines, were verified and completed, if appropriate. Bidirectional block was confirmed by differential pacing and/or repeat mapping, whenever linear lesions were generated. Acute, complete procedural success was defined as the termination of all stable, inducible AT, and the non-inducibility of further sustained tachycardias, confirmed by routine atrial burst pacing until a minimum cycle length of at least 30 ms below the AT cycle length or atrial refractoriness was reached [20].

### 2.5. Follow-Up

All patients were routinely followed-up at 3 months after the index procedure, with subsequent follow-ups every 3 to 6 months [20]. During follow-up, patients were left on their preprocedural antiarrhythmic medication. Pacemaker and implantable cardioverter defibrillators were interrogated through remote monitoring. 12-lead as well as 24 h Holter electrocardiograms were performed in all patients without implantable devices for further assessment. Arrhythmia recurrences were defined as episodes lasting >30 s and occurring >3 months after ablation or repeat ablation performed ≤3 months after ablation [21]. In the case of a recurrent, sustained, or symptomatic arrhythmia, patients were considered for a repeat ablation [22,23]. Types of arrhythmia recurrences were determined as AT, including typical atrial flutter, or AF. In addition to the arrhythmia mechanism, the origin of the clinical tachycardia and cycle length of the AT was analyzed. If the cycle length in the following procedures was in a range of ±20 ms of the index procedure’s AT cycle length and had the same origin as determined in the three-dimensional maps, the AT were considered similar [17].

### 2.6. Statistical Analysis

Continuous variables are presented as mean ± SEM or median (interquartile range: 25th–75th percentile) and were compared by Student’s *t*-test or Mann–Whitney U test, according to the normality of the distribution. Categorical variables are presented as counts (percentage) and were compared by Fisher’s exact test. Event-free survival was estimated using the Kaplan–Meier method and compared using the log-rank test. A *p* value < 0.05 was considered statistically significant. All analyses were performed using GraphPad Prism 10.4.1 (GraphPad Inc., La Jolla, CA, USA).

## 3. Results

### 3.1. Patient Characteristics

**Figure 1:**
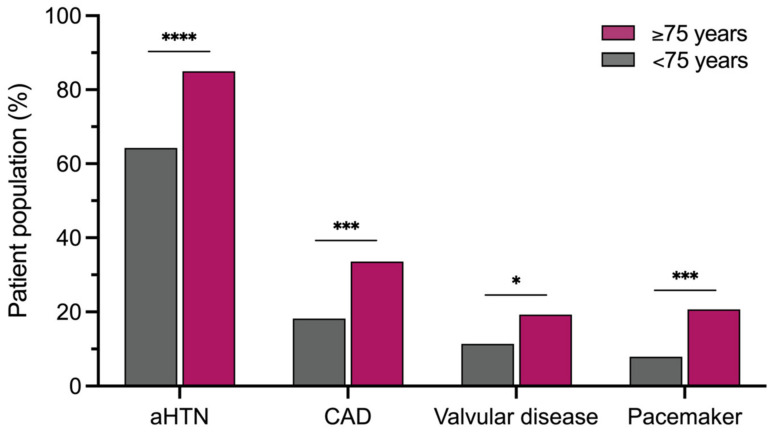
Elderly patients present with more cardiac comorbidities. Patients ≥75 years present more frequently with arterial hypertension, coronary artery disease, valvular disease, and with an implanted pacemaker compared to patients <75 years. aHTN = arterial hypertension; CAD = coronary artery disease. **** *p* < 0.0001; *** *p* < 0.001; * *p* < 0.05.

Patients ≥75 years (mean age 78.1 ± 0.2 years, 22.9% aged ≥80 years (range 80–86 years)) were more often female (54.3% vs. 38.2%; *p* = 0.0024) and had a lower body mass index (25.9 ± 0.4 kg/m^2^ vs. 26.9 ± 0.3 kg/m^2^; *p* = 0.0338) and a higher CHA_2_DS_2_-VA score (3 (IQR 3–4) vs. 2 (IQR 1–3); *p* < 0.0001) than patients <75 years (mean age 62.2 ± 0.6 years). They exhibited more cardiac comorbidities, including arterial hypertension (85.0% vs. 64.3%; *p* < 0.0001), coronary artery disease (33.6% vs. 18.2%; *p* = 0.0006), and valvular disease (19.3% vs. 11.4%; *p* = 0.0366). The latter mainly comprised moderate/severe mitral regurgitation in 15.0% vs. 9.3% (*p* = 0.0999). Additionally, they presented more often with an implanted device (26.4% vs. 12.9%, *p* = 0.0009), driven by a higher prevalence of pacemakers (20.7% vs. 7.9%; *p* = 0.0002) mainly due to sick sinus syndrome, but also third-degree atrioventricular block in some patients (Figure 1). Previous cardiac defibrillator implantation was performed in 5.7% vs. 5.0% (*p* = 0.8173) for primary/secondary prevention due to heart failure with reduced ejection fraction or after prior ventricular tachycardia/fibrillation. The prevalence of cardiomyopathy was not different between patients ≥75 and <75 years (16.4% vs. 15.7%; *p* = 0.8879). In patients ≥75 years, the underlying cardiomyopathy could be characterized as dilated (*n* = 5, 3.6%), hypertrophic (*n* = 4, 2.9%), ischemic (*n* = 2, 1.4%), valvular cardiomyopathy (*n* = 1, 0.7%), sarcoidosis (*n* = 1, 0.7%), takotsubo cardiomyopathy (*n* = 1, 0.7%), or tachymyopathy (*n* = 9, 6.4%). Groups did not differ regarding history of arrhythmia (98.7 ± 6.2 months vs. 98.7 ± 8.1 months; *p* = 0.9991), previous cardiac surgery (19.3% vs. 17.9%; *p* = 0.7892), or number of prior atrial ablation procedures (1.9 ± 0.1 vs. 2.1 ± 0.1; *p* = 0.2062). Among patients ≥75 vs. <75 years, previous atrial ablation involved pulmonary vein isolation in 87.1% vs. 86.4% (*p* = 0.8802), with additional substrate-based ablation in 38.6% vs. 42.1% (*p* = 0.5281). In total, 55.0% vs. 60.7% of patients underwent >1 prior ablation. Medication at admission, including antiarrhythmic drugs (31.4% vs. 38.6%; *p* = 0.1625) and beta-receptor blockers (78.6% vs. 80.7%; *p* = 0.6069), was comparable (Table 1).

### 3.2. Procedural Characteristics

**Figure 2:**
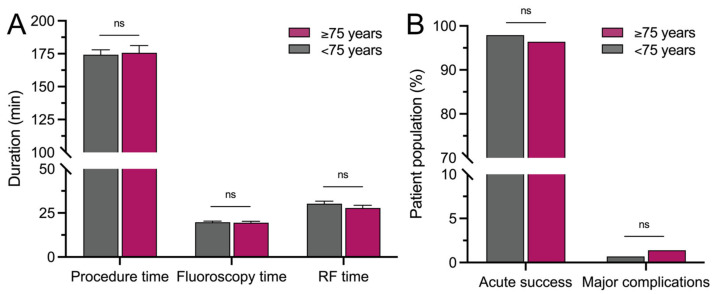
Procedural data, acute success, and major complications do not differ significantly between elderly and younger patients. (**A**) Procedural data and (**B**) acute procedural success and major complications do not differ significantly between patients ≥75 and <75 years. ns = not significant; RF = radiofrequency.

Procedural data, including procedure duration (175.7 ± 5.5 min vs. 174.2 ± 3.8 min; *p* = 0.8172), fluoroscopy duration (19.5 ± 0.8 min vs. 19.7 ± 0.7 min; *p* = 0.8650), radiofrequency application time (27.8 ± 1.6 min vs. 30.3 ± 1.4 min; *p* = 0.3033), and number of radiofrequency applications (28.9 ± 1.9 vs. 27.3 ± 1.4; *p* = 0.5284), were not different between patients ≥75 years and <75 years (Figure 2A). Similarly, applied energy did not differ (46,813 ± 2627 J vs. 50,709 ± 2464 J; *p* = 0.3792).

Comparing patients ≥75 years vs. <75 years, AT mechanisms were macro-reentrant in 61.9% vs. 65.8% (*p* = 0.4599), localized reentrant in 25.8% vs. 23.6% (*p* = 0.6380), and focal in 12.3% vs. 10.6% (*p* = 0.5174). Main AT locations were the anterior left atrial wall (18.7% vs. 14.4%; *p* = 0.2709), the left atrial roof (15.5% vs. 12.9%; *p* = 0.4676), and the mitral isthmus (7.1% vs. 10.6%; *p* = 0.2963).

Acute procedural success was reached in 96.4% vs. 97.9% of patients (*p* = 0.5173). The duration of hospital stays (2 (IQR 2–4) days vs. 2 (IQR 2–3) days; *p* = 0.9125) as well as the rate of complications (6.4% vs. 5.0%; *p* = 0.6496) did not differ. Major complications occurred in 1.4% vs. 0.7% of patients ≥75 years vs. <75 years (*p* = 0.6035), including pericardial effusion requiring pericardiocentesis (1.4% vs. 0%; *p* = 0.1106) and a cerebral embolic event with complete clinical recovery (0% vs. 0.7%; *p* = 0.5545) (Figure 2B). Detailed procedural characteristics are listed in Table 2. Pacemaker implantation following the unmasking of sick sinus syndrome was conducted in 1.4% vs. 1.1% (*p* > 0.9999).

Among all index procedures, the Rhythmia and the Carto mapping systems were used in 92.1% vs. 7.9%. Acute procedural success (97.4% vs. 97.0%; *p* = 0.5981) and major complications (0.8% vs. 3.0%; *p* = 0.2800) did not differ, depending on the system used.

### 3.3. Follow-Up

**Figure 3:**
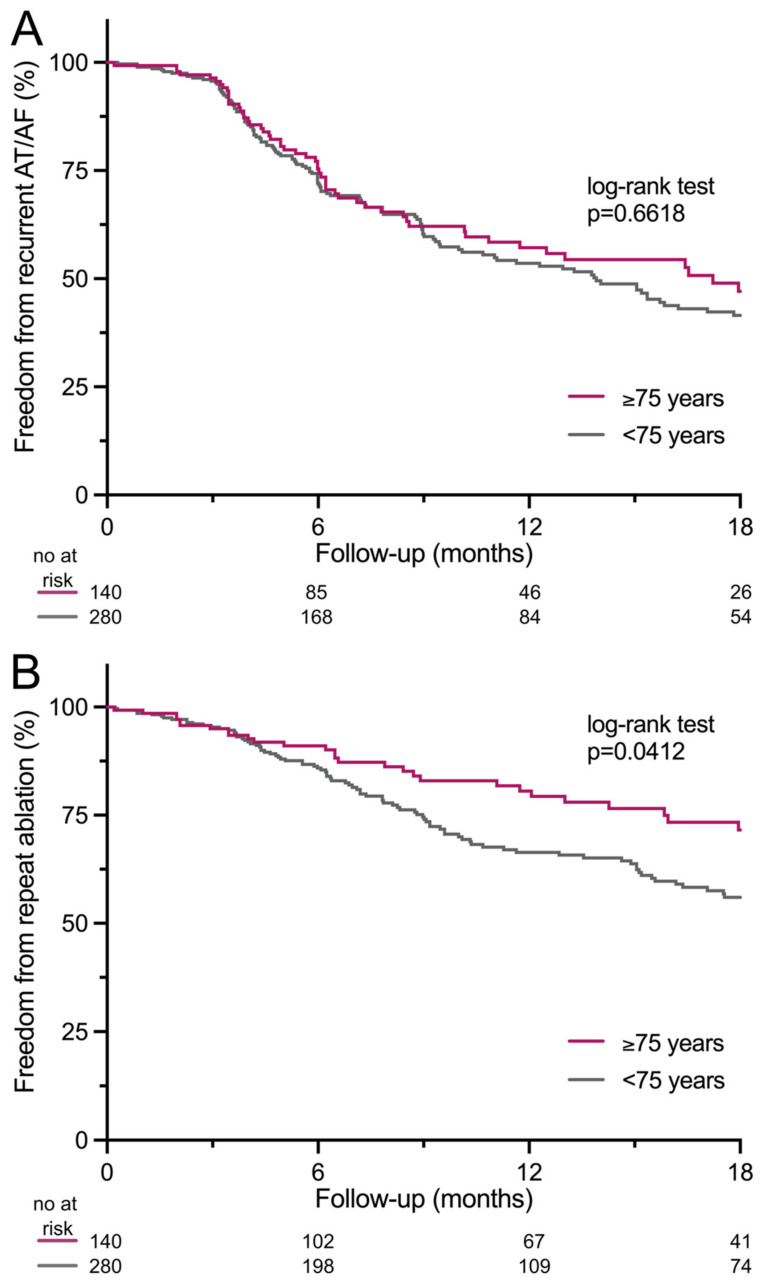
Repeat ablation is less frequently performed in elderly patients. Cumulative event-free survival probability curve illustrates freedom from (**A**) arrhythmia recurrence and (**B**) repeat ablation in patients ≥75 vs. <75 years. AF = atrial fibrillation; AT = atrial tachycardia. *p* values are based on the log-rank test.

During a follow-up of 364 (IQR 183–729.5) days, arrhythmia recurrences occurred in 45.0% vs. 49.3% of patients ≥75 years vs. <75 years (*p* = 0.4684) after 183 (IQR 118–357) vs. 182 (IQR 118.8–341.3) days (*p* = 0.8989) (Figure 3A). The percentage of recurrent AT (69.8% vs. 60.8%) or AF (30.2% vs. 39.2%) did not differ (*p* = 0.2617). Comparing patients undergoing index ablation with the Rhythmia or the Carto mapping system, 47.8% vs. 48.5% developed any sustained arrhythmia recurrence (*p* > 0.9999).

Repeat ablation was less frequently performed in patients ≥75 years (25.7% vs. 36.1%; *p* = 0.0361) after 260.5 (IQR 109.3–530.8) vs. 238 (IQR 132.5–449) days (*p* = 0.6375) (Figure 3B). The number of repeat ablation procedures (1.1 ± 0.03 vs. 1.3 ± 0.04; *p* = 0.0907), as well as the recurrence rate of the index AT (2.1% vs. 1.8%; *p* > 0.9999), did not differ between patients ≥75 years and <75 years (Table 3). Recurrence of the index AT was due to mitral isthmus-dependent AT (1.4% vs. 1.1%; *p* > 0.9999) or septal AT (0.7% vs. 0.7%; *p* > 0.9999).

## 4. Discussion

The present study investigated safety and outcomes of catheter ablation for consecutive AT in elderly patients ≥75 years after previous cardiac interventions. The main findings are that (1.) acute success and major complications do not differ significantly between patients ≥75 years and <75 years undergoing AT ablation and (2.) arrhythmia recurrences are comparable, but repeat ablation is less frequently performed in patients ≥75 years.

### 4.1. Impact of Age on Safety of AT Ablation

Aging is commonly associated with an increased risk of adverse cardiovascular outcomes, such as stroke, bleeding, and mortality, which significantly influences management in the elderly population in terms of pharmacological and interventional approaches for cardiac arrhythmias [14]. As for AF, as the most common cardiac arrhythmia, there have been conflicting results on the safety of catheter ablation in elderly patients [11,12,15,24,25,26]. A recent meta-analysis indicated that patients ≥75 years have an approximately 65% higher risk of procedural complications and safety endpoint occurrence compared to younger patients. Still, as the absolute risk of complications in older patients is only about 2% higher, clinical implications—especially regarding the potential of more standardized ablation approaches used nowadays—need to be investigated in detail [11]. Furthermore, in a different study, during a median follow-up of 3.5 years, there were no differences in all-cause hospitalization and mortality after AF ablation [26]. Similarly, in a cohort of patients undergoing ablation for typical atrial flutter, the risk of procedural complications was described to be almost 2-fold higher in older patients, mainly driven by an increased number of minor complications, as well as pacemaker implantations during follow-up. One reason may be that elderly patients presented more frequently with underlying heart diseases and tended to receive antiarrhythmic drugs and/or beta-receptor blockers more often [27]. In contrast, a more recent study confirmed similar complication rates in elderly compared to younger patients with typical atrial flutter, despite presenting with more comorbidities. However, it should be noted that 10% of the elderly patients subsequently underwent device implantation due to disturbed atrioventricular conduction or sick sinus syndrome and intended antiarrhythmic drug initiation for concomitant AF [28].

When transmitting these assumptions and observations to patients with AT, we now present first evidence that AT ablation can be safely performed in elderly patients with a similar risk of overall and major complications compared to a younger cohort, despite more comorbidities, including both classical risk factors and cardiac entities. As procedure durations of AT ablation are commonly longer due to often more extensive mapping and ablation with multiple co-existing tachycardias [29,30] and patient-related factors increasing clinical complexity, conscious systematic screening with potential modification of risk factors and periprocedural management of comorbidities is necessary to achieve comparable safety outcomes [14,31]. This may involve strict management of blood pressure and fluid balance, with possible usage of diuretics, especially in patients with heart failure, to avoid fluid overload. Furthermore, in patients with diabetes mellitus, periprocedural repeated glucose monitoring is of importance, whereas particularly in those with chronic renal insufficiency, minimization of contrast medium volume should be pursued to prevent contrast-associated acute kidney injury. Importantly, during left atrial procedures, effective intraprocedural heparinization should be targeted considering the high thromboembolic risk of this population, as confirmed in our study by an increased CHA_2_DS_2_-VA score [32]. Of interest, in our analysis, procedural data including applied energy did not differ between groups, emphasizing that usage of standard radiofrequency application times and energy levels additionally appears to be safe in elderly patients, while avoiding prolonged procedure and fluoroscopy times.

### 4.2. Impact of Age on Outcome of AT Ablation

Aging is associated with atrial fibrosis and regional conduction slowing, providing an altered arrhythmogenic substrate in the elderly population [4,33]. Whether this applies more for postmenopausal women than for men is understudied. However, the impact of estrogen and testosterone on atrial electrophysiology has been described, including potential beneficial effects of estrogen by prolongation of action potential duration and attenuation of fibrosis, as well as pro-arrhythmogenic effects of testosterone [34,35]. Elderly patients may more frequently exhibit distinct atrial fibrosis, whereas younger patients are commonly at an earlier disease stage and their arrhythmogenic substrate might still progress. This might explain the observation of a higher treatment effect of typical atrial flutter ablation in elderly compared to younger patients [27]. However, considering the high ablation success rate in this population, this difference might not show clinical relevance [28].

Catheter ablation has become the most effective therapy for AT to maintain sinus rhythm, with rapidly improving mapping and ablation technologies during the last decade [22]. Our analysis demonstrates similar acute as well as mid-term success rates in elderly patients ≥75 years compared to younger patients. Likewise, the recurrence rate of the index AT did not differ in our analysis. This suggests that currently employed ablation strategies efficiently target the critical AT site, despite a heterogeneous electrical substrate as a result of previous cardiac interventions, existing comorbidities, and/or structural heart diseases. Still, it should be noted that, in our study, repeat ablation was less frequently conducted in patients ≥75 years. This might be explained by a more conservative approach, which was probably pursued in this population considering that frailty and multiple comorbidities [36] might contribute to atrial structural remodeling and, therefore, impact long-term outcomes. Consequently, risk–benefit analysis in elderly patients with known advanced arrhythmogenic substrates—including atrial cardiomyopathy [37]—is necessary, taking into account arrhythmia burden and symptom severity, as well as concomitant clinical and procedural risk factors.

### 4.3. Limitations

Considering the study design, some limitations need to be addressed. First, this is a dual-center observational study of consecutive patients without a propensity-matched analysis, which resulted in a higher comorbidity burden in the elderly group. However, this observation represents daily practice, providing insights into patient characteristics and procedural planning. This approach further enables avoidance of selection bias for study inclusion, as all consecutive elderly patients undergoing AT ablation after previous cardiac interventions were investigated. Second, despite including elderly patients with multiple comorbidities, those at the highest risk might not be represented, as ablation therapy was declined or not feasible due to frailty. Third, since follow-up data did not consist of continuous electrocardiogram recordings in all patients, we have no conclusive information on asymptomatic recurrences. However, symptomatic arrhythmia recurrences in particular might be more meaningful for elderly patients, as they might lead to recurrent hospitalizations and reduced quality of life. Fourth, the present study may be underpowered to detect a difference in major complications given the small number of events, as there were numerically twice as many complications in patients ≥75 years vs. <75 years. Finally, patients ≥75 years of age undergoing AT catheter ablation in a tertiary arrhythmia center may represent a highly selective cohort, which should be considered during application in other populations.

## 5. Conclusions

Catheter ablation for consecutive AT in patients ≥75 years after previous cardiac interventions in tertiary arrhythmia centers is safe and effective. Acute and mid-term outcomes do not differ between patients ≥75 years and <75 years, but repeat ablation is less frequently performed. Therefore, AT ablation should not be ruled out in elderly patients due to age alone, but should be considered based on arrhythmia burden and symptom severity, as well as concomitant clinical and procedural risk factors.

## Figures and Tables

**Table 1:** Baseline characteristics of patients ≥75 vs. <75 years undergoing AT ablation.

Variable	Total (*n* = 420)	≥75 Years (*n* = 140)	<75 Years (*n* = 280)	*p* Value
Age, years	67.5 ± 0.5	78.1 ± 0.2	62.2 ± 0.6	-
Female sex Body mass index, kg/m^2^ Left ventricular ejection fraction, % CHA_2_DS_2_-VA score Arterial hypertension Coronary artery disease Cardiomyopathy Diabetes mellitus Stroke Valvular disease *Moderate/Severe mitral regurgitation* *Moderate/Severe aortic regurgitation* *Moderate/Severe aortic stenosis* *Moderate/Severe tricuspid regurgitation* Implantable device *Pacemaker* *Cardiac defibrillator* Previous cardiac surgery History of arrhythmia, months Previous atrial ablation Previous atrial ablations per patient Duration since last ablation, months Medication at admission Antiarrhythmic drugs *Class I* *Amiodarone* Beta-receptor blockers Oral anticoagulation	183 (43.6) 26.6 ± 0.2 54.7 ± 0.4 2 (1–3) 299 (71.2) 98 (23.3) 67 (16.0) 51 (12.1) 36 (8.6) 59 (14.0) *47* (*11.2*) *6* (*1.4*) *5* (*1.2*) *1* (*0.2*) 73 (13.4) *51* (*12.1*) *22* (*5.2*) 77 (18.3) 98.7 ± 5.8 398 (94.8) 2.1 ± 0.1 32.3 ± 1.8 152 (36.2) *63* (*15.0*) *68* (*16.2*) 336 (80.0) 385 (91.7)	76 (54.3) 25.9 ± 0.4 54.9 ± 0.8 3 (3–4) 119 (85.0) 47 (33.6) 23 (16.4) 20 (14.3) 13 (9.3) 27 (19.3) *21* (*15.0*) *4* (*2.9*) *2* (*1.4*) *0* (*0*) 37 (26.4) *29* (*20.7*) *8* (*5.7*) 27 (19.3) 98.7 ± 6.2 130 (92.9) 1.9 ± 0.1 36.3 ± 3.3 44 (31.4) *18* (*12.9*) *22* (*15.7*) 110 (78.6) 131 (93.6)	107 (38.2) 26.9 ± 0.3 54.7 ± 0.5 2 (1–3) 180 (64.3) 51 (18.2) 44 (15.7) 31 (11.1) 23 (8.2) 32 (11.4) *26* (*9.3*) *2* (*0.7*) *3* (*1.1*) *1* (*0.4*) 36 (12.9) *22* (*7.9*) *14* (*5.0*) 50 (17.9) 98.7 ± 8.1 268 (95.7) 2.1 ± 0.1 30.4 ± 2.1 108 (38.6) *45* (*16.1*) *46* (*16.4*) 226 (80.7) 254 (90.7)	0.0024 0.0338 0.8473 <0.0001 <0.0001 0.0006 0.8879 0.3456 0.7143 0.0366 *0.0999* *0.0986* *>0.9999* *>0.9999* 0.0009 *0.0002* *0.8173* 0.7892 0.9991 0.2061 0.2062 0.2974 0.1625 *0.4689* *0.8892* 0.6069 0.3551

Values are presented as mean ± SEM, median (IQR) or *n* (%).

**Table 2:** Procedural characteristics of patients ≥75 vs. <75 years undergoing AT ablation.

Variable	Total (*n* = 420)	≥75 Years (*n* = 140)	<75 Years (*n* = 280)	*p* Value
Procedure duration, min	174.7 ± 3.1	175.7 ± 5.5	174.2 ± 3.8	0.8172
Fluoroscopy duration, min Radiofrequency application time, min Radiofrequency applications Total energy, J Periprocedural AT per patient AT cycle length, ms Acute procedural success Duration of hospital stay, days Complications *Groin complication* *Pericardial effusion requiring pericardiocentesis* *Cerebral embolic event with complete clinical recovery*	19.7 ± 0.5 29.5 ± 1.1 27.8 ± 1.1 49,488 ± 1873 1.4 ± 0.04 305.0 ± 4.5 409 (97.4) 2 (2–4) 23 (5.5) *19* (*4.5*) *2* (*0.5*) *2* (*0.5*)	19.5 ± 0.8 27.8 ± 1.6 28.9 ± 1.9 46,813 ± 2627 1.5 ± 0.1 311.8 ± 8.3 135 (96.4) 2 (2–4) 9 (6.4) *7* (*5.0*) *2* (*1.4*) *0* (*0*)	19.7 ± 0.7 30.3 ± 1.4 27.3 ± 1.4 50,709 ± 2464 1.4 ± 0.04 301.6 ± 5.3 274 (97.9) 2 (2–3) 14 (5.0) *12* (*4.3*) *0* (*0*) *2* (*0.7*)	0.8650 0.3033 0.5284 0.3792 0.4519 0.2947 0.5173 0.9125 0.6496 *0.8045* *0.1106* *0.5545*

Values are presented as mean ± SEM, median (interquartile range) or *n* (%).

**Table 3:** Follow-up of patients ≥75 vs. <75 years undergoing AT ablation.

Variable	Total (*n* = 420)	≥75 Years (*n* = 140)	<75 Years (*n* = 280)	*p* Value
Arrhythmia recurrence	201 (47.9)	63 (45.0)	138 (49.3)	0.4684
Time to first recurrence, days	182 (118–345.5)	183 (118–357)	182 (118.8–341.3)	0.8989
Repeat ablation procedure	137 (32.6)	36 (25.7)	101 (36.1)	0.0361
Time to first repeat ablation procedure, days	239 (128–458)	260.5 (109.3–530.8)	238 (132.5–449)	0.6375
Number of repeat ablation procedures	1.2 ± 0.03	1.1 ± 0.03	1.3 ± 0.04	0.0907

Values are presented as mean ± SEM, median (interquartile) or *n* (%).

## Data Availability

The data underlying this article will be shared on reasonable request to the corresponding author.
